# Extracellular vesicles as biomarkers and modulators of atherosclerosis pathogenesis

**DOI:** 10.3389/fcvm.2023.1202187

**Published:** 2023-05-26

**Authors:** Sarvatit Patel, Mandy Kunze Guo, Majed Abdul Samad, Kathryn L. Howe

**Affiliations:** ^1^ Toronto General Hospital Research Institute, University Health Network, Toronto, ON, Canada; ^2^ Institute of Medical Science, University of Toronto, Toronto, ON, Canada; ^3^ Division of Vascular Surgery, Department of Surgery, University of Toronto, Toronto, ON, Canada; ^4^ Peter Munk Cardiac Centre, University Health Network, Toronto, ON, Canada

**Keywords:** extracellular vesicles, atherosclerosis, biomarkers, therapeutics, EV tracking models

## Abstract

Extracellular vesicles (EVs) are small, lipid bilayer-enclosed structures released by various cell types that play a critical role in intercellular communication. In atherosclerosis, EVs have been implicated in multiple pathophysiological processes, including endothelial dysfunction, inflammation, and thrombosis. This review provides an up-to-date overview of our current understanding of the roles of EVs in atherosclerosis, emphasizing their potential as diagnostic biomarkers and their roles in disease pathogenesis. We discuss the different types of EVs involved in atherosclerosis, the diverse cargoes they carry, their mechanisms of action, and the various methods employed for their isolation and analysis. Moreover, we underscore the importance of using relevant animal models and human samples to elucidate the role of EVs in disease pathogenesis. Overall, this review consolidates our current knowledge of EVs in atherosclerosis and highlights their potential as promising targets for disease diagnosis and therapy.

## 1. Introduction

Atherosclerosis is a significant cause of cardiovascular disease (CVD) that can lead to heart attack, stroke, kidney failure, and major amputation (1–4). Approximately 17.9 million people die from CVD annually (5). Atherosclerosis is a chronic inflammatory process characterized by endothelial activation, accumulation of lipoproteins, and recruitment of inflammatory cells that leads to plaques that gradually enlarge and either restrict blood flow or embolize, damaging the heart or peripheral tissues (6). The current diagnostic methods for atherosclerosis are associated with rare but significant procedure-related consequences and considerable cost (7, 8). The classical biomarkers, such as total cholesterol, low-density lipoprotein (LDL), or serum triglyceride levels, are the gold standard diagnostic tests for atherosclerosis (9). C-reactive protein, a non-specific inflammatory marker, has emerged as a clinical marker for residual risk in atherosclerosis patients with good cholesterol control (10, 11). Many of these biomarkers can diagnose CVD but cannot definitively predict stroke or myocardial infarction (MI) risk. There is a need for new CVD biomarkers that are cost-effective, improve detection, and identify novel treatment targets. As we enter the era of precision medicine, we need a more granular understanding of biomarkers that can be used as reliable screening tools with metrics to guide personalized intervention to prevent devastating clinical events.

**Figure 1:**
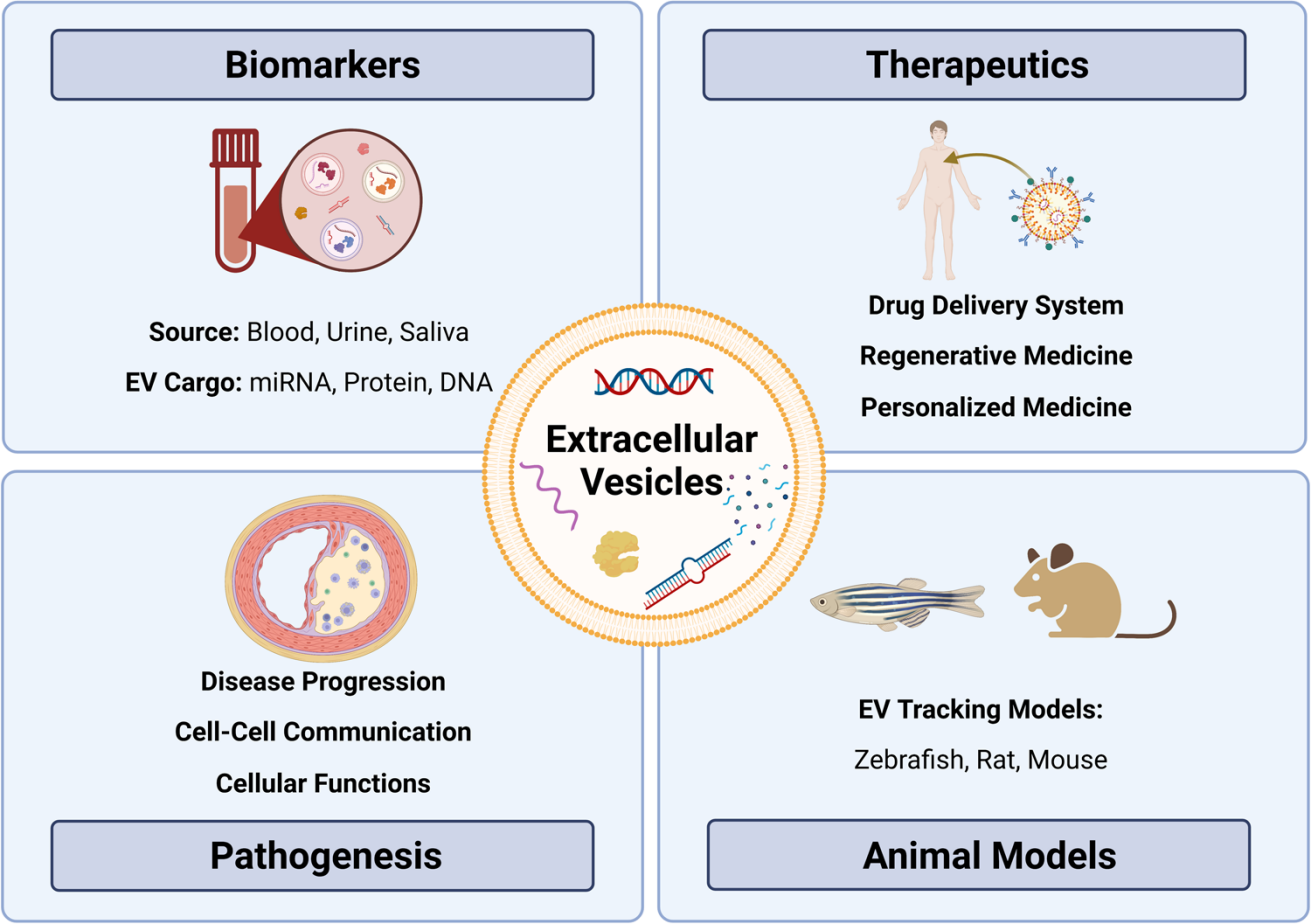
Exploring the role of extracellular vesicles in atherosclerosis: insights from biomarkers, therapeutics, pathobiology, and translational models. Extracellular vesicles (EVs) as versatile entities for various applications. EVs, small membrane-bound particles, have emerged as promising biomarkers for diagnostic and prognostic purposes in various diseases. They have also shown great potential as therapeutic agents for their ability to carry and deliver bioactive molecules. Moreover, EVs have been implicated in the pathogenesis of many diseases, including cancer, neurodegenerative diseases, and atherosclerosis. Animal studies have contributed significantly to our understanding of the biology and functions of EVs, paving the way for their clinical translation.

The American Heart Association proposed seven metrics in 2010 to define and monitor cardiovascular health (12). Managing the disease involves non-pharmacological methods (healthy diet, regular physical activity, and tobacco abstinence) (1) and pharmacological interventions such as statins to control lipoprotein levels (13–15), with newer options such as cholesterol-binding agents (e.g., ezetimibe) (16) and proprotein convertase subtilisin/kexin type 9 (PCSK9, lowers LDL) inhibitors (e.g., evolocumab) (17–19) also available. Notably, several studies have highlighted challenges in achieving therapeutic goals for serum lipids despite high-intensity statin therapy (20–22). In some cases, surgery or stent-based therapies are required to manage more severe atherosclerosis. While current strategies can slow the progression of atherosclerosis and/or prevent clinical events (23), further research is needed to understand the specific cellular and molecular mechanisms underpinning plaque progression to identify targets for stabilization and/or plaque regression. One area of promise includes delineating cellular communication during atherosclerotic plaque development and progression. In this regard, extracellular vesicles (EVs) have been identified as essential cell-cell communicators that may hold promise in improving our understanding of atherosclerotic disease—from biomarkers to disease pathogenesis (24–26) (Figure 1).

## 2. Extracellular vesicles

### 2.1. Biogenesis, cargo, and functions

EVs are lipid bilayer-bound particles that all cell types release into the extracellular space. They can be classified into three major types based on their biogenesis, morphological, and biochemical properties: exosomes (form as intraluminal vesicles within multivesicular bodies that fuse with the plasma membrane, 30–100 nm), microvesicles (directly bud off from healthy plasma membrane/also referred to as ectosomes, 100–1,000 nm) and apoptotic bodies (form during apoptosis, 1–5 µm) (27, 28). Furthermore, EVs carry cargo that contains biologically active materials, such as DNA, microRNA, messenger RNA, proteins, lipids, and carbohydrates. Once released into the extracellular space, EVs may directly interact with nearby cells (28). EVs can enter biological fluids *via* transcytosis or by breaching biological barriers, where they can travel throughout the body *via* the circulation—either blood or lymphatics (28, 29). EVs can then be taken up by recipient cells *via* endocytosis, fusion with the recipient cell plasma membrane, or binding to target cell membrane proteins (30, 31). The transferred cargo to recipient cells can affect molecular and cellular signalling pathways and functions.

Current EV isolation permits classification based on size, density and surface markers but does not discriminate based on biogenesis (28). That said, proteomic analysis has revealed distinct protein composition for EV subtypes (32), with some markers helping to distinguish EVs by biogenesis pathways. For example, exosome markers include endosomal sorting complexes required for transport (ESCRT) proteins, Alix and tetraspanins, while ectosome markers include Annexin A2/A5, ARF6 and Enolase 1 (32). Although advanced technology will undoubtedly yield more discrimination between EV populations, some promise exists in using inhibition of EV biogenesis by pharmacological therapies. For example, inhibitors of cancer exosome secretion may impact cancer progression and metastasis (33). Ultimately, the ideal strategy will be to find specific inhibitors that can impact EVs associated with pathology but not those that play critical physiological roles (34). To do this, we will need a more nuanced understanding of the kinetics of EV release from the host cell, travel within the circulation, recipient cell uptake, and EV clearance.

EV cargo is biologically active. In cancer, EV cargo can promote neoplastic transformation and cell proliferation, contributing to cancer initiation and progression (35–38). During atherogenesis, EVs released from endothelial cells (ECs) and immune cells promote leukocyte infiltration and plaque maturation (39–41). This suggests that EVs circulating in the plasma could serve as non-invasive disease biomarkers. Both *in vitro* and *in vivo* studies have shown that circulating EVs carry microRNA, which can be biomarkers for neurodegenerative diseases (42–45) and CVD (46). EVs possess several unique advantages compared to traditional biomarkers (47). EVs are stably circulated in almost all bodily fluids, they can represent the current disease state by carrying specific cargo from parental cells, and they can be collected sequentially. As a result, EVs have significant potential as clinically valuable biomarkers capable of providing multiple, minimally intrusive assessments of the disease state.

EVs can also be used as a stable drug delivery system that protects cargo from degradation (48–54). EVs have numerous advantages over cell-based therapies in regenerative medicine, such as long shelf life, ease of transportation, long-term storage, and lack of replication (55–57). As drug delivery vehicles, they outperform synthetic drug carriers by crossing tissue and cellular barriers (48). In preclinical studies, EVs have been used as a drug delivery system. For instance, exosome-mediated siRNA delivery has been used in Alzheimer's disease (45), while mesenchymal stem cell-derived exosomes have been used to treat ischemic lung injury (58) and eye disorders (59). However, further understanding of EV circulation dynamics, targeting, internalization, and intracellular trafficking pathways is needed to fully capitalize on the therapeutic potential.

### 2.2. Characterization

EV isolation is divided into three main approaches based on size, density, and surface markers (31). Size-exclusion chromatography (SEC) is one commonly used technique for EV isolation based on size, while differential ultracentrifugation exploits the distinct density gradients of EVs (60). Finally, magnetic beads/affinity chromatography or flow cytometry uses surface markers to extract EVs with high specificity but low yield (31). There is no single gold standard. Although ultracentrifugation has been widely used in the past, there has been a shift towards SEC attributed in part to the higher EV yield and functionality obtained through SEC (61, 62).

The Minimal Information for Studies of Extracellular Vesicles (MISEV) 2018 provides a key tool for standardizing EV research (31) and has helped to establish rigour in a rapidly emerging field of research by outlining criteria for EV quantification and characterization. Suggestions include EV quantification by nanoparticle tracking analysis (NTA) (63), characterization by surface marker protein expression using western blot (31), and purity control to detect the presence of non-vesicular contamination, such as apolipoprotein A1 and albumin in EVs enriched from plasma (64). Imaging EVs *via* electron microscopy is recommended (63), while flow cytometry detecting surface markers can be used to characterize the cellular origin of EVs (65, 66). In this way, rigorous determination of cell-specific EVs holds promise as highly specific biomarkers for a disease state. EVs can be further characterized by analyzing their cargo. Mass spectrometry has been used to study EV proteomics in biofluids and tissues (67, 68). Similarly, transcriptomics has been employed to investigate the nucleic acid cargo of EVs, specifically microRNA cargo, primarily through microarrays and RT-qPCR, which are limited to a particular RNA panel (69).

Despite the considerable advancement in technology for EV isolation and characterization, limitations and challenges remain. However, as developing technology continues to refine EV research, it is becoming clear that EVs play crucial roles in biological processes, govern disease, and have emerged as a new avenue in atherosclerosis research.

## 3. EVs in atherosclerosis

### 3.1. EVs in plasma

EVs might serve as diagnostic and therapeutic tools for many CVD conditions. EV levels in the blood, urine and saliva have been linked to clinical risk in patients with stable CVD (70) (Table 1). Elevated EVs are associated with risk factors such as smoking, diabetes, and hypercholesteremia (84). The abundance of EVs carried in plasma reflects the potential for utilizing these EVs as biomarkers for CVD, and notably that EVs derived from specific cell types, such as ECs, leukocytes and platelets, correlate with CVD (85). A previous study exploited the surface markers expressed on EVs to purify and isolate cell-specific EVs, followed by enrichment and analysis of EV cargo. In EVs isolated from plasma, CD14 upregulation was linked to a higher risk of ischemic stroke occurrence (80), while increased cystatin C and polygenic immunoglobin receptors were linked to acute coronary syndrome (77).

**Table 1:** Potential biomarkers for cardiovascular diseases.

Disease	Sample	Biomarker	Levels	Study Design	Potential application	Reference
Heart Failure (HF)	Plasma	miR-1254, miR-1306	↑	2203 patients with HF	Prognosis	(71)
		Galectin-3	↑	1329 patients with HF	Prognosis	(72)
	Saliva	Galectin-3	↑	64 patients with HF; 51 healthy controls	Diagnosis	(73)
	Plasma-EVs	miR-425, miR-744	↓	31 patients with HF; 31 healthy controls	Diagnosis	(74)
	Serum-EVs	mir-92B-5P	↑	28 patients with HF; 30 healthy controls	Diagnosis	(75)
Acute Coronary Syndrome (ACS)	Plasma	miR-208b, miR-133a	↑	444 patients with ACS	Diagnosis	(76)
	Serum-EVs	pIgR, cystatin C	↑	471 ACS-suspected patients	Diagnosis	(77)
Coronary Artery Disease (CAD)	Urine	collagen α1 (I and III)	↑	67 patients presenting with symptoms suspicious for CAD	Diagnosis	(78)
	Plasma-EVs	miR-126, miR-199a	↑	181 patients with stable CAD	Prognosis	(79)
Vascular Disease	Plasma-EVs	Cystatin C, Serpin F2, CD14	↑	1060 patients with vascular disease and severe vascular risk factors	Prognosis	(80)
Myocardial Infarction (MI)	Plasma	miR-499-5p, miR-208b	↑	424 patients with suspected ACS	Prognosis	(81)
	Serum-EVs	miR-192, miR-194, miR-34a	↑	21 patients with MI; 65 matched controls	Prognosis	(82)
Atherosclerosis	Plasma	PIGR, IGHA2, APOA, HPT, HEP2	↑	222 patients with atherosclerosis; 222 matched controls	Prognosis	(83)

Circulating EVs from different cellular origins and their distinct cargo (e.g., microRNA, protein) have been linked to pathological conditions such as dyslipidemia, diabetes (86–88), CVD (60, 79), and inflammatory disorders (89–91). This suggests that EVs play a role in the immune response, vascular remodelling, endothelial dysfunction, and apoptosis, all of which underlie atherosclerosis (92, 93). Studies have shown that leukocyte-derived, neutrophil-derived, and activated platelet-derived EVs were significantly higher in patients with atherosclerosis (94, 95). EVs carried in plasma may be helpful as biomarkers for atherosclerosis, but accuracy must be improved to detect changes in EV count from specific cell types. As EVs are heterogeneous in size, composition, and cellular origin, it makes identifying specific populations and correlating them to disease challenging (46). In addition to EV heterogeneity, clinical variables (e.g., age, sex), comorbidities (e.g., obesity), and clinical history (e.g., cancer, medications) affect circulating EVs in plasma (31).

Furthermore, laboratory standardization will be critical before employing EVs as a biomarker: lack of standard protocols for EV isolation, quantification, and characterization leads to variability in results and negates their utility as a biomarker or clinical assessment tool. EV biomarkers lack a standard reference range, making it difficult to compare among populations and studies. No EV-based biomarker has been adopted for CVD, and more research is needed to develop standard protocols to study plasma EVs. Work is ongoing, as the International Society for Extracellular Vesicles has a specific blood task force focused on standardizing plasma and serum-derived EVs (96).

To address the challenges in EV detection, standardization, and clinical translation, technological improvements, standardized protocols, and prospective large clinical trials are needed (97, 98). Precision medicine EV research has recently become more prominent. It is a potential path that enables physicians and researchers to use patient data to develop personalized treatments. For instance, a multi-biomarker approach may incorporate EV evaluation for screening/diagnosis, prognosis, and monitoring of people at risk of atherosclerotic CVD (97). Several subsets of EV biomarkers can be exploited for patient risk evaluations, reclassification, and disease stage diagnosis (97). Applying transcriptomic and proteomic analysis plus artificial intelligence algorithms to clinical data can help identify high-risk individuals and administer preventive strategies quickly (98).

### 3.2. EVs in plaque

As EVs protect their molecular cargo from degradation and carry surface markers identifying their parent cell, plasma EVs a unique opportunity to study disease states (diagnostic potential). On the other hand, EVs in tissue can contribute to the pathophysiology or progression of plaques (therapeutic targets) (99). At this time, however, screening and tracing EVs released from cells or tissues *in vivo* remains challenging. EVs are found in early and advanced plaques, suggesting they are involved in both the initiation and advanced phases of plaque development in humans (100–102). Patients with atherosclerosis demonstrated enrichment of proatherogenic EV cargo, such as vascular cell adhesion molecule-1, von Willebrand Factor, endothelial nitric oxide synthase, and angiopoietin-1, compared to healthy control participants (103, 104). Athough EV production, function, and quantity in atherosclerotic lesions still needs more delineatation, some granularity is emerging, with human atherosclerotic plaques containing EVs derived from leukocytes, macrophages, erythrocytes, lymphocytes, and smooth muscle cells (SMCs) (101, 102, 105).

Most of our current understanding of the role of EVs in atherosclerosis has been obtained from studies using EVs derived from cell cultures, which may not accurately represent EVs found *in vivo*. Nonetheless, EVs have been found to exert significant influence over a range of pro-atherogenic processes, such as inflammation, thrombosis, and angiogenesis (84). In particular, EC-derived EVs have been implicated in endothelial dysfunction (106, 107) and vascular inflammation (108), which may contribute to the development of early atherosclerotic plaques. Moreover, EC-derived EVs can communicate with macrophages (109, 110) and SMCs (111–113) to regulate vascular disease, while monocyte-derived EVs have been found to modulate vascular inflammation and cell death (114–116). Additionally, foam cell-derived EVs have been shown to regulate SMC migration, thereby potentially accelerating the progression of atherosclerotic lesions (117). EVs produced from a range of cell types, including T-cells, platelets, dendritic cells, and monocytic cells, have also been shown to cause macrophage apoptosis (118–122), that may contribute to the development and progression of atherosclerosis. Moreover, EVs have been found to play various multifaceted and environment-dependent roles in other cellular processes, such as endothelial permeability (123), pro- and anti-inflammatory signaling (124–126), leukocyte transmigration and lipid accumulation (127–129), SMC proliferation (130), intravascular calcification (131), extracellular matrix remodeling (132), and plaque rupture. Collectively, the evidence suggests a substantial role for EVs in atherosclerosis pathogenesis, emphasizing the need for additional research into the mechanisms underlying EV-mediated intercellular communication in this disease. New technologies such as flow cytometry and single EV analysis will increase detection precision and provide more detail on cell-specific EV phenotypes, their cargo, and their role in disease regulation. Until then, an emerging resource for EV studies is the development of multicellular models for tracking.

## 4. EV tracking animal models

Despite recent discoveries, it is still challenging to understand the spatiotemporal distribution and physiological activities of EVs *in vivo*. Little is known about the biological activities of EVs *in vivo*, including tissue distribution, blood levels, and clearance dynamics. EVs have been investigated in several disease-simulating animal models, including mouse, rats, and zebrafish.

The transparent nature of zebrafish makes them an ideal model (133, 134). The transgenic zebrafish model was recently established, enabling in vivo identification, tracking, and isolation of endogenous EVs produced by different cell types (135). A cell membrane-tethered fluorophore reporter system in the zebrafish allows cell-specific EV tracking and the potential to track EVs in cell-cell communication within the cardiovascular system (135). *In vivo*, a live EV tracking model of zebrafish demonstrated inter-organ communication by endogenous exosomes (136). Despite these successes, zebrafish EV tracking models have technological limitations (136) and consequently, more complex models are needed.

EVs have also been studied using more advanced organisms for models of diseases such as cancer (137, 138) and neurological disorders (139–141). For example, a murine model was used to determine the therapeutic effects of immunity and matrix regulatory cell-derived EVs on idiopathic pulmonary fibrosis (142). Several studies have used the rat model to study the role of EVs in spinal cord injuries (143, 144) and repetitive stress (145). Rat models have also been used to investigate the therapeutic potential of EVs to treat diseases such as small cerebral vessel disease (146), colo-cutaneous post-surgical fistula (147), and congenital diaphragmatic hernia (148).

EVs and their relevance to various disease processes have been investigated over the past decade, but EV tracking *in vivo* remains challenging. Understanding EV biodistribution throughout an organism will be essential before use in clinical practice. Using an EV tracking mouse model, a few studies have demonstrated EV-mediated cell-cell communication and the effects of EVs produced from tumour cells on distant organs (149–151). Investigators used Cre-LoxP mouse models to study cell or tissue-specific EVs by crossing CD9/CD63-GFPf/f exosome reporter mice with Cre-mice (*α*MHC-MerCreMer for cardiomyocytes, Pax8-Cre for renal tubular epithelial cells, Cdh5-CreERT2 for ECs, villin-Cre for intestine, and alb-Cre for liver) (152–154). In addition, a transgenic rat model (GFP-tagged human CD63) was employed to determine intercellular and mother-to-child EV transfer *in vivo* (155). However, a complete understanding of the role of cell-specific EVs in atherosclerotic diseases remains elusive. To investigate the potential of EVs as biomarkers or disease modulators, novel animal models that allow for the tracking, characterization, and evaluation of cell-specific EVs are required.

## 5. Perspectives for future studies

EVs, known to carry biologically active cargoes, appear to play an important role in the pathogenesis of atherosclerosis. However, studying plaque-derived EVs is challenging due to limited accessibility and the complex composition of plaques. Thus, researchers have turned to studying EVs in circulation, particularly in those carried in plasma, to gain insights into atherosclerosis biology. Although plasma is easily accessible, identifying reliable biomarkers is challenging, and pairing biomarkers with clinical events may not reflect the disease state entirely. Therefore, it is necessary to determine whether disease regions release EVs into circulation, which could serve as a potential biomarker. The paired assessment of EVs from circulation and plaques of the same patient is one possible approach, representing a promising and meaningful strategy for an atherosclerosis study.

Further research must elucidate the precise mechanisms by which plaque-derived EVs contribute to atherosclerosis pathogenesis to identify potential therapeutic targets. The primary challenge in studying EVs within the plaque is determining their source (cell-specific EVs) and their functions on neighbouring cells. Transgenic zebrafish models have demonstrated the feasibility of tracking EVs within the cardiovascular system (135). More sophisticated animal models are essential to enable EV-tracking into plaques, better comprehend EV biogenesis and metabolism, and investigate cell-specific EV roles and functions during disease progression. Plasma-derived EVs (e.g., leukocyte origin) have demonstrated potential as biomarkers for assessing plaque vulnerability in patients (156, 157). The potential for EVs carried in plasma to shed light on the biology of atherosclerotic plaque vulnerability to rupture (leading to clinical events such as MI and stroke) is promising and requires further investigation. Using EVs as therapeutic targets for atherosclerosis is a growing area of interest, given their stability as drug-delivery vehicles (158, 159). Overall, EVs represent a promising area for future research in the field of atherosclerosis.

## 6. Conclusions

EVs have been recognized as important components in the pathogenesis of atherosclerosis. EVs derived from immune and ECs are implicated in developing and destabilizing atherosclerotic plaques. Plasma EVs carry the potential for conveying information related to the vulnerability of atherosclerotic plaques and can serve as potential biomarkers for atherosclerosis and its associated complications, such as MI and stroke. Moreover, the prospect of utilizing EVs as therapeutic targets for atherosclerosis has recently gained substantial interest. While work remains to improve the tools and standardization of EV research, EVs nonetheless represent an encouraging area for future research in the field of atherosclerosis and hold the potential to provide novel insights into the diagnosis, treatment, and prevention of this chronic inflammatory disease.
